# Impacts of Fog Characteristics, Forward Illumination, and Warning Beacon Intensity Distribution on Roadway Hazard Visibility

**DOI:** 10.1155/2016/4687816

**Published:** 2016-05-22

**Authors:** John D. Bullough, Mark S. Rea

**Affiliations:** Lighting Research Center, Rensselaer Polytechnic Institute, Troy, NY 12180, USA

## Abstract

Warning beacons are critical for the safety of transportation, construction, and utility workers. These devices need to produce sufficient luminous intensity to be visible without creating glare to drivers. Published standards for the photometric performance of warning beacons do not address their performance in conditions of reduced visibility such as fog. Under such conditions light emitted in directions other than toward approaching drivers can create scattered light that makes workers and other hazards less visible. Simulations of visibility of hazards under varying conditions of fog density, forward vehicle lighting, warning beacon luminous intensity, and intensity distribution were performed to assess their impacts on visual performance by drivers. Each of these factors can influence the ability of drivers to detect and identify workers and hazards along the roadway in work zones. Based on the results, it would be reasonable to specify maximum limits on the luminous intensity of warning beacons in directions that are unlikely to be seen by drivers along the roadway, limits which are not included in published performance specifications.

## 1. Introduction

Individuals in the transportation, construction, and utility sectors are overrepresented in workplace fatalities in the US [1]. Flashing yellow warning beacons are important components of worker safety for these workers. It is estimated that in US roadway construction work zones alone, more than 100 workers are killed annually in work related accidents [2]. To the extent that these beacons provide warning and information to drivers approaching them, these devices will contribute to transportation, construction, and utility worker safety.

A number of studies have been undertaken to ascertain the necessary peak intensity and flashing characteristics of warning beacons to ensure rapid and accurate detection [3–8]. Peak intensity of a beacon, primarily in the horizontal direction, is most important for detection by drivers along the roadway, and most performance standards for warning beacons only specify performance at and near this horizontal direction [9–11]. This direction is probably most critical for drivers' detection of the warning beacons from distances of at least a hundred meters away. The amount of light directed in other directions, particularly upward, is not included in current warning beacon performance standards.

In clear weather conditions, light emitted upward by warning beacons is, at worst, wasted because it is not likely to reach the eyes of nearby drivers who, presumably, would adjust their driving behavior upon detecting a flashing warning beacon. However, during perturbed atmospheric conditions, such as fog, upward intensity is not merely wasted but it will contribute to deleterious scattered light in the atmosphere [12]. This scattered light will reduce the contrast, and therefore the visibility, of front line service workers, both at fixed locations and around moving service vehicles such as snow plows and construction vehicles. The magnitude of the scattered light depends not only on the intensity of the warning beacon but also on the proximity of the warning beacon to the observer's line of sight in the field of view; scatter superimposed over objects further from the warning beacon will be lower compared to that over objects close to the warning beacon [13].

Luminance contrast reductions are relevant to driving safety because visual performance is related not only to the absolute light level and the size of the object to be seen, but also to the contrast between an object and its background. By reducing the contrast of objects in the field of view, scattered light can result in complete loss of object detection or recognition as well as longer visual response times to those objects [15]. Scattered light in fog will be particularly detrimental for low contrast and small objects already near the visibility threshold. Moreover, older drivers will be most affected due to reductions in the optical fidelity of the aging eye.

The presence of fog has been found in several studies to have a number of empirical safety-related impacts. Kang et al. [16] found that headway distance decreased and the likelihood of misjudging a preceding vehicle's speed increased as the density of fog increased. Cavallo et al. [17], Buchner et al. [18], and Broughton et al. [19] also found that increased fog density resulted in reduced headway distances to preceding vehicles. Among the reasons given by these authors for changes in headway and misjudgments of speed is the reduced visual performance caused by scattered light in fog.

Despite these potential impacts, warning beacon performance standards [9–11] do not specifically address their performance for different ambient conditions, such as snow or fog, when scattered light and glare can affect the appearance and effectiveness of the warning beacons. In order to assess impacts of upward warning beacon intensity distribution on visibility of front line workers or of potential hazards in and along the roadway, a physically accurate model of light propagation in fog was used to assess the amount of scattered light in a simulated driving scene. The absolute luminous intensity and the distribution of intensity of the simulated warning beacon were varied along with fog density and the amount of forward illumination from a vehicle's headlights. Using a model of visual performance [15], the visibilities of two targets, one close to and one further away from the simulated warning beacon, for the different simulations were determined. A physically accurate model of light propagation in fog was used for these analyses because, even under strictly controlled conditions, the optical properties of real or artificial fog can vary widely [20, 21].

Using the results of these simulations, visual performance analyses were conducted to assess the impacts of the factors described above on the ability of drivers of different ages to respond to potential hazards in and along the road. The objective of these analyses is to identify whether, and if so, how, existing performance standards for warning beacons should be modified when used in inclement weather conditions such as nighttime fog.

## 2. Method

### 2.1. Simulation Software

A series of roadway scenarios was created using the simulation software package (PROF: Photometric Rendering of Fog), developed by Dumont [22] at the Laboratoire Central des Ponts et Chaussées (LCPC), now the Institut Français des Sciences et Technologies des Transports de l'Aménagement et des Réseaux (IFSTTAR). The PROF software uses Monte Carlo ray tracing [23] to model the dispersion of light among surfaces and through atmospheric conditions defined by the user. Monte Carlo ray tracing has been found by Battistelli et al. [24], by Dutré et al. [25], and by Girasole et al. [26] to provide a physically accurate model of the optical properties of fog and other perturbed atmospheric conditions. In Monte Carlo analysis, individual rays of light are modeled stochastically. Light can either reach a simulated surface in the modeled scene, leave the physical boundary limits of the modeled scene, or be scattered by the atmospheric medium. Dumont [23] demonstrated that Monte Carlo simulation closely matched physical measurements of light distributions in fog conditions when at least 10 million rays were traced.

### 2.2. Simulation Scenarios

In each scenario, a warning beacon was represented by a point source having an isotropic luminous intensity of either 150 cd or 750 cd. These values represent the likely range of warning beacon luminous intensities that might be experienced by drivers, based on research findings [3, 4, 6, 7] and on published performance specifications [9–11]. The warning beacon was located 100 m ahead of a simulated viewing location, which corresponds approximately to the stopping distance for an unalerted driver at a speed of 80 km/h [27, 28], and 3 m above the ground surface, typical for a mounting height on road construction equipment [29]. The ground was modeled as a Lambertian surface with a reflectance of 0.1 [30], similar to that of asphalt pavement. The simulation software does not model the complex reflectance distributions of surfaces such as asphalt, so a Lambertian distribution was chosen. When a specular surface was specified in the simulation, a mirror image of the warning beacon appeared in the horizontal pavement surface between the beacon and observer locations, with a luminous intensity proportional to the reflectance of the surface.

Three warning beacon intensity distributions were modeled: the warning beacon could be totally* unshielded* so that it produced the same luminous intensity in all directions around the beacon, it could be shielded by a perfect black* baffle* 1 m in front of the beacon that absorbed all light at and above the horizontal direction, or it could produce a* beam* angle of 10° with maximum intensity in the direction of the observer. For all intensity distributions, the luminous intensity in the direction of the simulated viewing location, which was located 100 m in front of the warning beacon location and 1.1 m above the ground [31], was either 150 cd or 750 cd. The simulated viewing direction for the observer was a point at ground level directly below the warning beacon, 100 m ahead of the observer location.

**Figure 1:**
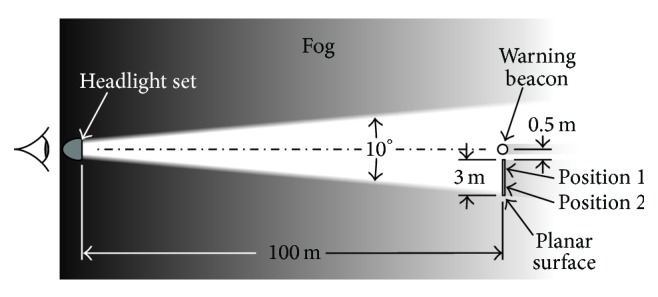
Plan view layout for the modeled scenarios (not to scale).

A planar surface adjacent to the simulated warning beacon was also included in the simulation. This surface could be analytically delineated into different shapes (e.g., pedestrian silhouette) and different areas (e.g., near to or further from the warning beacon) to simulate potential hazards on the roadway. The surface was modeled as a 2 m (high) × 3 m (wide) rectangle perpendicular to the ground plane with a Lambertian reflectance of 0.1, typical of dark clothing worn by most pedestrians [32]. A simulated automobile headlight set was positioned 0.6 m above the ground and 100 m from the simulated warning beacon and adjacent planar surface. The headlights had a beam angle of 10° with an isotropic luminous intensity of either 30,000 cd or 100,000 cd, representing typical luminous intensities from low- and high-beam headlamps [33].  Figure 1 shows a plan view of the simulation.

The simulation software requires the scenario geometry to be specified in an input text file from the perspective of the observer. The observer's viewing direction is at the center of a 200 (vertical) × 320 (horizontal) rectangular array subtending 28° and 45°, respectively.

The atmosphere conditions and the number of rays to be used in the simulation must also be defined in the input text file. The fog particle diameters in each simulation ranged from about 1 *μ*m to 10 *μ*m and varied approximately as a logarithmic Gaussian distribution with a mean size between 3 and 4 *μ*m [34]. Each simulation assumed a fog scattering function approximated by the Henyey-Greenstein model [21]. This model uses a scattering coefficient *g* that represents the relative amount of forward scattering or backscattering (−1 is all backscattering; +1 is all forward scattering). A value of 0.8 was used for *g* in the model scenarios, representing mostly forward scattering, which is representative of most advection and radiation fogs [21]. In comparison, typical smoke-filled atmospheres exhibit greater backscatter and have a value for *g* of approximately 0.3 [35]. Based upon Dumont [23], who traced 10 million rays for accurate results, 100 million rays were traced for each simulation in the present study.

Four fog densities were used, corresponding to meteorological visibility distances of 50 m, 200 m, and 600 m and infinity (no fog). A distance of 50 m corresponds to the borderline between dense and thick fog, 200 m corresponds to the borderline between thick and moderate fog, and 600 m corresponds to the borderline between thin and moderate fog conditions [36]. The range between 50 m and 200 m is thought to be of the most relevance to safety for highway driving applications [37]; at lower visibility distances, driver visibility is extremely impaired and at greater distances impacts on visibility are relatively small [37]. Spectral selectivity was not considered in the simulations. Light scatter in fog is largely insensitive to wavelength [12, 13, 38] because the suspended water droplets are large relative to the wavelengths of light. For simulated clear conditions (i.e., no fog), only a single warning beacon intensity distribution (*unshielded*) was used for each peak intensity, since there would be no atmospheric scatter difference among the distributions.

### 2.3. Visual Performance Analyses

**Figure 2:**
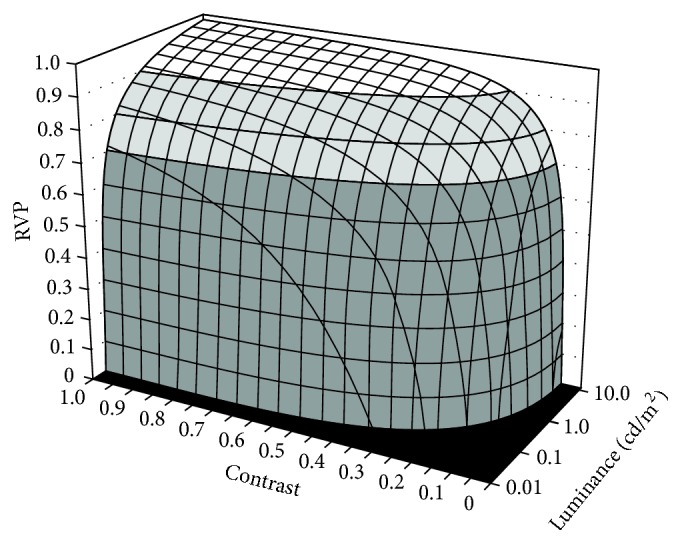
RVP values for a standard 20 cm × 20 cm visibility target [14] viewed from 46 m as a function of background luminance and contrast. An RVP value of 0 corresponds to the threshold for object identification; negative values (not shown) are possible for objects that can be detected but not identified. RVP values less than 0.8 are considered unsatisfactory for driving safety because small reductions in target contrast or target size will render potential hazards invisible.

The visibilities of pedestrian targets (located at Positions 1 and 2 in Figure 1) were determined for each modeled fog scenario using the relative visual performance (RVP) model developed by Rea and Ouellette [15]. The RVP model was derived from experimental data obtained from subjects performing a numerical verification task and validated by subjects performing a reaction time task. Each set of psychophysical experiments used a wide range of light levels, target sizes, and target contrasts. RVP is a unitless quantity ranging from 0 at the threshold of identification to values of 1 or higher for large, high-contrast visual targets under high light levels. Negative values of RVP occur when an object can be detected, but not identified. RVP exhibits a plateau characteristic whereby once visual performance results in high accuracy and short response times (i.e., RVP ≥ 0.8), further increases in light level, size, or contrast will yield diminishing performance benefits in comparison to the resources (e.g., higher light levels) required to achieve them.  Figure 2 illustrates the functional relationship between RVP and combinations of background luminance and target contrast for a standard small (20 cm × 20 cm, 0.5 reflectance) target [14] viewed from 46 m.

RVP is nearly always on the plateau for interior applications such as offices, but this is not the case for outdoor applications such as roadway and vehicle lighting [39, 40], making RVP a sensitive measure of visual performance for roadway lighting conditions. A number of studies of visual responses under nighttime driving conditions have been conducted including sign legibility [41, 42], pedestrian identification times [43, 44], and stopping distances to roadside hazards [45, 46]. In each case, measured response times and detection distances were strongly correlated with RVP quantities determined by experimental conditions. Further, increases in RVP associated with the presence of roadway intersection lighting have been demonstrated to be strongly correlated with reductions in nighttime crash frequency associated with intersection lighting [47]. Taken together, this body of previous research demonstrates the utility of RVP as a meaningful safety-related measure of visual performance in nighttime driving situations [48].

The luminance of the simulated pedestrian targets and that of the immediate background were used to calculate their luminance contrasts for locations closer to and further from the warning beacon's angular location (Positions 1 and 2, resp., in Figure 1). In addition to luminance veils produced by scattered light in the fog volume for each scenario, veiling luminance from entoptic scattered light in the eye that contributes to disability glare [49] was also included in the visual performance analyses. Using a visual detail size of 0.1 m^2^, which corresponds to the critical visual detail size for detecting pedestrians [50] and a viewing distance of 100 m, RVP values were calculated for a 60-year-old driver under each scenario. This age corresponds approximately to the 80th percentile for driver ages in the US [51].

## 3. Results and Discussion

**Figure 3:**
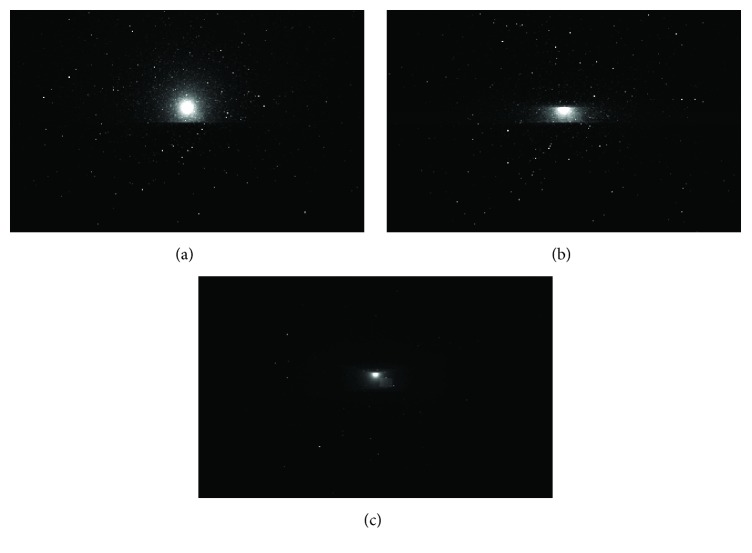
Roadway scene renderings corresponding to low-beam forward illumination (30,000 cd), a fog visibility range of 600 m, a warning beacon intensity of 750 cd, and each spatial intensity distribution ((a) unshielded; (b) baffle; (c) beam). Each panel is an eight-bit (0–255) rendering; a pixel value of 0 (black) corresponds to a luminance of 0 cd/m^2^ and a pixel value of 255 (white) corresponds to a luminance of 2.55 cd/m^2^ or higher. The actual photometric simulations did not truncate the higher luminance levels.

Table 1 summarizes the target and background luminance, luminance contrasts, and RVP values for each simulation. As an example, renderings of the luminance arrays for the simulations corresponding to low-beam forward illumination (30,000 cd), 600 m visibility range, and a warning beacon intensity of 750 cd and for each spatial intensity distribution (*unshielded*,* baffle*, and* beam*) are shown in Figure 3. The decrease in the luminance veil produced by scattered light in fog as the intensity distribution narrows is evident from visual observation of each panel of Figure 3. As the warning beacon luminous intensity distribution changes from* unshielded*, to* baffle*, and to* beam*, the luminance contrast of the target in Position 1 (closer to the warning beacon) increases, from 0.13, to 0.14, and to 0.26, respectively, and the RVP value increases from 0.328, to 0.386, and to 0.647, respectively.

## 4. Conclusions

Flashing yellow warning beacons are intended to alert drivers to potential hazards such as workers or parked vehicles in work zones and similar locations. Warning beacons should not, however, impair drivers' visibility of those potential hazards. Scattered light from warning beacons in perturbed atmospheric conditions, such as fog, can reduce the contrast of hazards, making them more difficult or impossible to see. Since no studies have been conducted on the impact of warning beacons in perturbed atmospheric conditions, the present study was aimed at assessing the impact of flashing warning beacons under different fog scenarios using a physically accurate model of scattered light characteristics in perturbed atmospheres. The primary findings of the present study were as follows.

To minimize contrast reduction of potential hazards near warning beacons, these light sources should be equipped with optics that limit light propagation in directions other than that toward the drivers that need to be warned. Table 1 shows that a directional beam (with a 10° beam angle) minimizes scattered light relative to an omnidirectional beacon. Optical control directing light toward oncoming drivers is also a more efficient use of electrical energy (affecting battery life) because all of the light emitted by the beacon is useful for purposes of warning drivers of potential hazards. It should be noted, however, that the optics of the warning beacon probably should ensure that low-level light is emitted in all directions to ensure the beacon can be seen by everyone near it.

The luminous intensity of the warning beacons should be reduced during fog and, logically, during other perturbed atmospheric conditions such as falling snow [38]. Assuming that the warning beacon can itself be seen, Table 1 shows that visibility (RVP) was always greater in fog for the low warning beacon intensity (150 cd) than for the high warning beacon intensity (750 cd).

It is interesting to note that the headlight intensity associated with high-beam operation (100,000 cd) was better for seeing hazards near the warning beacon than during low-beam operation (30,000 cd) except in the densest fog (50 m range). This is due to the dominant forward scattering properties of fog. In the densest fog evaluated, however, visibility is so low that neither low-beam nor high-beam operation will enable drivers to see potential hazards at 100 m, although the contrast of the targets was lower under high-beam illumination than under low beams. For comparison to an atmosphere exhibiting greater backscatter (such as smoke) with a scattering coefficient value for *g* of 0.3 [35] rather than 0.8 [21], the backscattered luminance from the low- and high-beam sources was calculated for both values of *g* and for visibility ranges of 50, 200, and 600 m (Table 2). Decreasing the value of *g* increases the backscattered luminance; the luminance for *g* = 0.3 at a visibility distance of 600 m is similar in magnitude to those for *g* = 0.8 at a visibility distance of 50 m. These data suggest that there are visibility penalties to driving at night with high beams in smoke and other atmospheres with greater backscatter than fog, even when the overall atmospheric density is relatively low.

**Figure 4:**
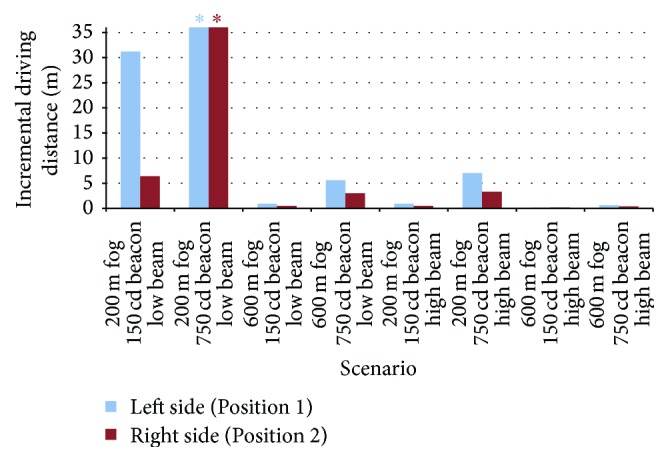
Increased vehicle driving distances associated with increases in hazard target visibility as the warning beacon was changed from the* unshielded* to the* beam* distribution, for several modeled fog scenarios. Asterisks (*∗*) indicate that the hazard target was invisible for the* unshielded* distribution, but the increase in luminance contrast with the* beam* distribution would substantially increase the resulting stopping distance.

Since RVP values and visual response times are inversely proportional [15, 48], it is possible to estimate the incremental distance that would be driven (assuming, for example, a driving speed of 80 km/h) because of the longer response times associated with poorer visual performance in the presence of the unshielded beacon relative to the narrow-distribution beacon. These incremental distances range from negligible (0.1 m) when the target was already visible even in the presence of the warning beacon (because of thin fog and high forward illumination) to as much as 31 m (see Figure 4). For some conditions (e.g., with a 750 cd beacon in fog with a visibility range of 200 m), the target was invisible in the presence of an unshielded warning beacon, but detectable for the beam distribution. This would correspond to a substantial increase in stopping distance even greater than 31 m but cannot be quantified precisely.

The reduced driving distances estimated as an impact of the warning beacon intensity distribution can have meaningful safety impacts [52], especially given the reduced headway that frequently occurs when driving in fog [16–19]. For this reason, it is reasonable to specify maximum limits on the luminous intensity of warning beacons in directions that are unlikely to be seen by drivers along the roadway, limits which are not included in published performance specifications [9–11].

As lighting and electronics become more sophisticated and less expensive, it is important to speculate on possible technologies that would improve the visual conditions associated with warning beacons. As noted previously in this section, it is important to reduce beacon intensity in fog so that hazards near the beacon can be seen more clearly. “Intelligent” systems that control beacon intensity overall as well as directional intensities in the presence of fog can be readily envisioned and, thus, engineered. These systems may be informed by manual control by people in a work zone or driving a utility vehicle, or automatically from weather reports or local feedback using a photosensor designed to detect backscatter from the beacon. The cost of these “intelligent” beacons would obviously be higher than those presently available, but a simple cost-benefit analysis suggests that a reduction of worker fatalities by 5% (i.e., avoiding 5 worker fatalities annually) over current annual levels could justify an incremental cost of $100 per beacon [2, 53–55] in work zones where workers are particularly exposed or where fog conditions are most common.

## Figures and Tables

**Table 1:** Hazard target luminance, contrast, and RVP value for each modeled scenario, assuming an observer age of 60 years. Negative values of RVP correspond to hazard targets that could be detected but not identified; the lowest RVP values were truncated to a value of −2.0.

Forward light source intensity (cd)	Fog visibility range (m)	Warning beacon intensity (cd)	Warning beacon distribution	Position 1: target luminance (cd/m^2^)	Position 1: luminance contrast	Position 1: RVP	Position 2: target luminance (cd/m^2^)	Position 2: luminance contrast	Position 2: RVP
30,000	50	150	Unshielded	0.65	0.07	<−2.0	0.64	0.08	<−2.0
			Baffle	0.64	0.08	<−2.0	0.64	0.08	<−2.0
			Beam	0.63	0.09	<−2.0	0.63	0.09	<−2.0
		750	Unshielded	0.78	0.06	<−2.0	0.72	0.07	<−2.0
			Baffle	0.74	0.07	<−2.0	0.70	0.07	<−2.0
			Beam	0.64	0.08	<−2.0	0.64	0.08	<−2.0
	200	150	Unshielded	0.65	0.09	<−2.0	0.59	0.12	−0.525
			Baffle	0.64	0.09	<−2.0	0.58	0.12	−0.496
			Beam	0.50	0.12	−0.849	0.49	0.14	−0.155
		750	Unshielded	1.58	0.04	<−2.0	1.23	0.06	<−2.0
			Baffle	1.52	0.04	<−2.0	1.20	0.06	<−2.0
			Beam	0.86	0.07	<−2.0	0.73	0.10	−1.479
	600	150	Unshielded	0.53	0.32	0.689	0.44	0.39	0.717
			Baffle	0.49	0.35	0.701	0.42	0.40	0.721
			Beam	0.37	0.46	0.738	0.35	0.49	0.745
		750	Unshielded	1.36	0.13	0.328	1.01	0.17	0.509
			Baffle	1.26	0.14	0.386	0.93	0.18	0.542
			Beam	0.65	0.26	0.647	0.55	0.31	0.683
	*∞*	0	Any	0.22	0.99	0.823	0.22	0.99	0.823
		150	Any	0.20	0.76	0.763	0.20	0.77	0.766
		750	Any	0.23	0.68	0.760	0.23	0.69	0.763

100,000	50	150	Unshielded	3.11	0.02	<−2.0	3.11	0.02	<−2.0
			Baffle	3.11	0.02	<−2.0	3.10	0.02	<−2.0
			Beam	3.10	0.02	<−2.0	3.09	0.02	<−2.0
		750	Unshielded	3.24	0.02	<−2.0	3.19	0.02	<−2.0
			Baffle	3.20	0.02	<−2.0	3.17	0.02	<−2.0
			Beam	3.12	0.02	<−2.0	3.10	0.02	<−2.0
	200	150	Unshielded	1.63	0.13	0.462	1.57	0.15	0.530
			Baffle	1.61	0.13	0.467	1.56	0.15	0.532
			Beam	1.48	0.15	0.512	1.47	0.16	0.559
		750	Unshielded	2.56	0.08	−0.021	2.21	0.10	0.295
			Baffle	2.49	0.09	0.027	2.18	0.11	0.307
			Beam	1.83	0.12	0.382	1.71	0.13	0.484
	600	150	Unshielded	1.27	0.46	0.861	1.19	0.50	0.866
			Baffle	1.24	0.48	0.863	1.17	0.50	0.867
			Beam	1.11	0.53	0.869	1.10	0.54	0.871
		750	Unshielded	2.11	0.28	0.818	1.76	0.34	0.838
			Baffle	2.01	0.29	0.824	1.68	0.35	0.842
			Beam	1.40	0.42	0.855	1.30	0.46	0.861
	*∞*	0	Any	0.67	0.99	0.901	0.67	0.99	0.901
		150	Any	0.67	0.79	0.880	0.66	0.80	0.881
		750	Any	0.70	0.71	0.873	0.69	0.72	0.873

**Table 2:** Backscattered luminance caused by headlights for different headlight source intensities, visibility ranges, and scattering coefficients.

Forward light source intensity (cd)	Visibility range (m)	Scattering coefficient (*g*)	Backscattered luminance (cd/m^2^)
30,000	50	0.3	4.57
		0.8	0.58
	200	0.3	2.17
		0.8	0.36
	600	0.3	0.87
		0.8	0.13

100,000	50	0.3	15.32
		0.8	3.03
	200	0.3	7.27
		0.8	1.18
	600	0.3	2.96
		0.8	0.46

## References

[1] National Institute for Occupational Safety and Health (2015). *The National Occupational Research Agenda*.

[2] Pegula S. (2013). An analysis of fatal occupational injuries at road construction sites, 2003–2010. *Monthly Labor Review*.

[3] Howard J., Finch D. M. (1960). Visual characteristics of flashing roadway hazard warning devices. *Highway Research Board Bulletin*.

[4] Hargroves R. A. (1971). A survey of the use of flashing lights on roads and road vehicles. *The Perception and Application of Flashing Lights*.

[5] Flannagan M. J., Blower D. F., Devonshire J. M. (2008). *Effects of Warning Lamp Color and Intensity on Driver Vision*.

[6] Bullough J. D., Rea M. S. Luminous intensity requirements for service vehicle warning beacons.

[7] Bullough J. D., Rea M. S. Warning beacon characteristics for visibility, glare prevention and closure detection.

[8] Gibbons R. B., Lee S. E., Williams B., Miller C. C. (2008). *Selection and Application of Warning Lights on Roadway Operations Equipment*.

[9] Society of Automotive Engineers (1990). *Flashing Warning Lamps for Authorized Emergency, Maintenance and Service Vehicles*.

[10] Society of Automotive Engineers (1997). *Optical Warning Devices for Authorized Emergency, Maintenance and Service Vehicles*.

[11] Society of Automotive Engineers (1998). *Gaseous Discharge Warning Lamp for Authorized Emergency, Maintenance and Service Vehicles*.

[12] Middleton W. (1952). *Vision through the Atmosphere*.

[13] Boelter L., Ryder F. (1940). Notes on the behavior of a beam of light in fog. *Illuminating Engineering*.

[14] Illuminating Engineering Society (2014). *Roadway Lighting, RP-8-14*.

[15] Rea M. S., Ouellette M. J. (1991). Relative visual performance: a basis for application. *Lighting Research and Technology*.

[16] Kang J. J., Ni R., Andersen G. J. (2008). Effects of reduced visibility from fog on car-following performance. *Transportation Research Record*.

[17] Cavallo V., Colomb M., Doré J. (2001). Distance perception of vehicle rear lights in fog. *Human Factors*.

[18] Buchner A., Brandt M., Bell R., Weise J. (2006). Car backlight position and fog density bias observer-car distance estimates and time-to-collision judgments. *Human Factors*.

[19] Broughton K. L. M., Switzer F., Scott D. (2007). Car following decisions under three visibility conditions and two speeds tested with a driving simulator. *Accident Analysis and Prevention*.

[20] Colomb M., Hirech K., André P., Boreux J. J., Lacôte P., Dufour J. (2008). An innovative artificial fog production device improved in the European project ‘FOG’. *Atmospheric Research*.

[21] Dumont E., Hautière N., Gallen R. (2010). A semi-analytic model of fog effects on vision. *Atmospheric Turbulence, Meteorological Modeling and Aerodynamics*.

[22] Dumont E. (2008). *Photometrical Rendering of Fog: Technical Documentation*.

[23] Dumont E. (1999). Semi-Monte Carlo light tracing applied to the study of road visibility in fog. *Monte Carlo and Quasi-Monte Carlo Methods*.

[24] Battistelli E., Bruscaglioni P., Ismaelli A., Zaccanti G. (1985). The effect of a strongly inhomogeneous medium on the propagation of light beams under multiple scattering conditions. *Optica Acta*.

[25] Dutré P., Lafortune E., Willems Y. Monte Carlo light tracing with direct computation of pixel intensities.

[26] Girasole T., Rozé C., Maheu B., Gréhan G., Ménard J. (1998). Visibility distances in a foggy atmosphere: comparisons between lighting installations by Monte Carlo simulation. *Lighting Research and Technology*.

[27] Olson P. L., Clevland D. E., Fancher P. S., Schneider L. W. (1984). *Parameters Affecting Stopping Sight Distance*.

[28] Jones E. R., Childers R. L. (2000). *Contemporary College Physics*.

[29] Safronov A. I. (1975). New construction and road machines of the Caterpillar company. *Hydrotechnical Construction*.

[30] Illuminating Engineering Society (1984). *Lighting Handbook: Reference Volume*.

[31] Sivak M., Flannagan M. J., Budnik E. A., Flannagan C. C., Kojima S. (1996). *The Locations of Headlamps and Driver Eye Positions in Vehicles Sold in the USA*.

[32] Bhise V. D., Farber E. I., Saunby C. S., Troell G. M., Walunas J. B., Bernstein A. (1998). Modeling vision with headlights in a systems context. *Motor Vehicle Lighting*.

[33] Akashi Y., Hu F., Bullough J. D. (2008). *Sensitivity Analysis of Headlamp Parameters Affecting Visibility and Glare*.

[34] Dumont E., Paulmier G., Lecocq P., Kemeny A. Computational and experimental assessment of real-time front-lighting simulation in night-time fog.

[35] Hawkins T., Einarsson P., Debevec P. (2005). Acquisition of time-varying participating media. *Computing Machinery Transactions on Graphics*.

[36] Meteorological Office College (1997). *Source Book to the Forecaster's Reference Book*.

[37] Shepard F. D. (1996). *Reduced Visibility Due to Fog on the Highway: Synthesis of Highway Practice*.

[38] Bullough J. D., Rea M. S. (2001). Driving in snow: effect of headlamp color at mesopic and photopic light levels. *Lighting Technology Developments for Automobiles*.

[39] Rea M. S. (1989). Visibility criteria and application techniques for roadway lighting. *Transportation Research Record*.

[40] Rea M. S. (2012). The Trotter Paterson Lecture 2012: whatever happened to visual performance?. *Lighting Research and Technology*.

[41] Goodspeed C. H., Rea M. S. (1999). The significance of surround conditions for roadway signs. *Journal of the Illuminating Engineering Society*.

[42] Schnell T., Yekhshatyan L., Daiker R. (2009). Effect of luminance and text size on information acquisition time from traffic signs. *Transportation Research Record*.

[43] Bullough J. D., Rea M. S., Zhang X. Evaluation of visual performance from pedestrian crosswalk lighting.

[44] Bullough J. D., Skinner N. P. Vehicle lighting and modern roundabouts: implications for pedestrian safety.

[45] Bullough J. D., Skinner N. P. Predicting stopping distances under different types of headlamp illumination.

[46] Skinner N. P., Bullough J. D. Influence of intelligent vehicle headlamps on pedestrian visibility in roundabouts.

[47] Bullough J. D., Donnell E. T., Rea M. S. (2013). To illuminate or not to illuminate: roadway lighting as it affects traffic safety at intersections. *Accident Analysis and Prevention*.

[48] Bullough J. D., Radetsky L. C. Roadway lighting, relative visual performance and safety.

[49] Fry G. A. (1954). A re-evaluation of the scattering theory of glare. *Illuminating Engineering*.

[50] Zhang X. (2009). *Evaluation of Approaches to Crosswalk Lighting Design*.

[51] Federal Highway Administration (2000). *Our Nation's Highways: 2000*.

[52] Sivak M., Flannagan M. J., Sato T., Traube E. C., Aoki M. (1994). Reaction times to neon, LED, and fast incandescent brake lamps. *Ergonomics*.

[53] U.S. Department of Transportation (2008). *Treatment of the Economic Value of a Statistical Life in Departmental Analyses*.

[54] Cook S., Quigley C., Clift L. (1999). Motor vehicle and pedal cycle conspicuity. *Vehicle Mounted Warning Beacons*.

[55] US Census Bureau (2009). *Statistical Abstract of the United States*.

